# Abnormal Expression of Prostaglandins E2 and F2*α* Receptors and Transporters in Patients with Endometriosis

**DOI:** 10.1155/2015/808146

**Published:** 2015-07-09

**Authors:** Halima Rakhila, Nathalie Bourcier, Ali Akoum, Marc Pouliot

**Affiliations:** ^1^Centre Hospitalier Universitaire de Québec, Québec, Canada G1V 4G2; ^2^Faculté de Médecine, Université Laval, Québec, Canada G1V 0A6

## Abstract

*Objective.* To investigate the level of expression of prostaglandin receptivity and uptake factors in eutopic and ectopic endometrium of women with endometriosis. *Design.* Prospective study. *Setting.* Human reproduction research laboratory. *Patients.* Seventy-eight patients with endometriosis and thirty healthy control subjects. *Intervention(s).* Endometrial and endometriotic tissue samples were obtained during laparoscopic surgery. *Main Outcome Measure(s).* Real-time polymerase chain reaction assay of mRNA encoding prostaglandin E2 receptors (EP1, EP2, EP3, and EP4), prostaglandin F2*α* receptor (FP), prostaglandin transporter (PGT), and multidrug resistance-associated protein 4 (MRP4); immunohistochemical localization of expressed proteins. *Results.* Marked increases in receptors EP3, EP4, and FP and transporters PGT and MRP4 in ectopic endometrial tissue were noted, without noticeable change associated with disease stage. An increase in EP3 expression and decreases in FP and PGT were observed in the eutopic endometrium of endometriosis patients in conjunction with the phases of the menstrual cycle. *Conclusion(s).* This study is the first to demonstrate a possible relationship between endometriosis and enhanced prostaglandin activity. In view of the wide range of prostaglandin functions, increasing cell receptivity and facilitating uptake in endometrial tissue could contribute to the initial steps of overgrowth and have an important role to play in the pathogenesis and symptoms of this disease.

## 1. Introduction

Endometriosis is a major health issue affecting nearly 10 percent of women of childbearing age. The main symptoms of this disease include chronic pelvic or abdominal pain, irregular bleeding, and in 40–50% of cases infertility [1]. The amount of pain experienced correlates poorly with disease stage. Endometriotic tissue may settle and proliferate in the fallopian tubes or the ovaries or enter the peritoneal cavity and deposit in ectopic sites. The causes and symptoms of endometriosis are multifactorial. Although knowledge of its underlying immunological and endocrine mechanisms is progressing, gray areas continue to obscure complete understanding of its pathology. Our studies were among the first to highlight dysfunctions in eutopic endometrium, including elevated levels of the monocyte chemoattractant factor MCP-1 [2]. In addition, we have shown that ectopic endometrial tissue by itself is capable of producing growth-promoting molecules such as vascular endothelial growth factor (VEGF) [3] as well as implantation-promoting integrins [4] while initiating peritoneal inflammation. This inflammation causes the release of mediators such as prostaglandins E2 (PGE2) and F2*α* (PGF2*α*), which play a role in reproductive functions such as ovulation, luteolysis, implantation, parturition, and lactation. Excessive release of prostaglandins may affect peritoneal function, causing pain [5] and disrupting processes such as oocyte maturation, ovulation, and fertilization [6, 7].

Prostaglandins are lipid compounds derived enzymatically from arachidonic acid. The reproductive system is the principal producer of PGE2 and PGF2*α*. The involvement of prostaglandins in pain [5, 8, 9], infertility [10–12], angiogenesis [13, 14], tissue remodeling [15], and cell proliferation [16] in association with various pathological conditions is well documented. We along with others have shown that an isoform of cyclooxygenase-2 (COX-2) is overexpressed in ectopic endometrial cells [17–20] and that PGE2, PGF2*α*, and other specific prostaglandins are present at abnormally high levels in uterine tissues of women suffering from menorrhagia, dysmenorrhoea, or endometriosis [21–23]. It is also well known that PGE2 and PGF2*α* are more concentrated in the peritoneal fluid of endometriosis patients [5, 10, 12, 24, 25]. Such evidence has led us to investigate a possible role of these prostaglandins in the pathogenesis of endometriosis. Our recent comparison of endometrium (eutopic and ectopic) from endometriosis patients to healthy eutopic endometrium showed overproduction of PGE2 and PGF2*α*, apparently promoted by increased expression of enzymes such as COX-2 with cPGES or AKR1C3 [26].

Regulation of production is not the only means by which the body modulates the action of PGE2 and PGF2*α*. Expression of prostaglandin receptors and transporters may also be regulated. Once released as messenger molecules, prostaglandins act locally on receptors in an autocrine or paracrine manner. The four main subtypes of PGE2 receptor are designated as EP1, EP2, EP3, and EP4, while the PGF2*α* receptor is called FP [27, 28]. The receptor subtype determines the nature of the physiological response. Reception either elicits the intracellular calcium-inositol triphosphate pathway or increases/decreases cyclic adenosine monophosphate (cAMP) activity. Engagement of some receptors may elicit both pathways, depending on cell type and receptor splice variety.

Prostaglandins were originally believed to exit from producer cells via passive diffusion because of their strongly lipophilic character. The discovery of the prostaglandin transporter protein PGT (SLCO2A1), which mediates prostaglandin uptake and release [29, 30], demonstrated that diffusion alone did not explain the penetration of prostaglandins through the cell membrane. Furthermore, a specific transporter, namely, multidrug resistance protein 4 (MRP4, ABCC4) of the ATP-binding cassette transporter superfamily, has been shown to mediate prostaglandin release [31]. Whether or not MRP4 is the only transporter that does this is still unclear.

Although it is clear that PGE2 and PGF2*α* play important roles in a number of female reproductive physiological processes as well as in endometriosis-associated infertility and pain [5, 10, 12, 32], current understanding of these roles remains incomplete. In the present study, we analyzed the expression of EP1, EP2, EP3, EP4, FP, PGT, and MRP4 in endometriosis patients in comparison to their expression in normal eutopic endometrium. We observed marked differences between eutopic and ectopic endometria in terms of prostaglandins receptivity and transport readiness.

## 2. Materials and Methods

### 2.1. Patients and Tissue Collection

The study received approval from the Human Research Ethics Committee at Saint-François d'Assise Hospital, and informed consent was obtained from all participants, who were recruited between February 2002 and March 2007. Endometriosis patients were aged 34.2 ± 3.6 years (*n* = 78) and were consulting for pelvic pain and/or infertility. They were diagnosed using laparoscopy and the disease stage (I–IV) was determined according to the Revised American Fertility Society classification system. Endometriotic tissue samples were collected from 28 of these patients. We also recruited healthy women aged 35.3 ± 3.8 (*n* = 30) scheduled for tubal ligation. These participants had no pelvic pathology or sign of endometrial hyperplasia or neoplasia and had not received any anti-inflammatory or hormonal medication for at least 3 months. Menstrual cycle dating was determined using the cycle history.

Endometrial and endometriotic biopsies were obtained during laparoscopy. Tissue was placed immediately at 4°C in sterile Hank's balanced salts solution (HBSS) (GIBCO Invitrogen Corp., Burlington, ON, Canada) containing 100 IU/mL penicillin, 100 *μ*g/mL streptomycin, and 0.25 *μ*g/mL amphotericin and transported to the laboratory. After washing in HBSS at 4°C, samples were frozen at −80°C in Eppendorf tubes for quantitative real-time PCR (qRT-PCR) or embedded in paraffin and stored at room temperature for immunohistochemical analysis.

### 2.2. Quantitative Real-Time PCR

Total RNA was extracted from endometrial tissue using the TRIzol reagent according to the manufacturer's instructions (Invitrogen Life Technologies, Inc., Grand Island, NY, USA) and reverse-transcribed in the presence of random hexamers. The qRT-PCR reaction was carried out in an ABI 7000 Thermal Cycler (Applied Biosystems, Foster City, CA, USA). The standard reaction mixture contained 2 *μ*L of RT product, 0.5 *μ*L of each primer (final concentration, 0.1 mM), 12.5 *μ*L SYBR Green PCR Master Mix (Invitrogen Life technologies, Inc., Grand Island, NY, USA) consisting of Taq DNA polymerase reaction buffer, dNTP mix, SYBR green I, MgCl_2_, and Taq DNA polymerase. Following denaturing for 2 min at 95°C, the reactions were cycled 45 times with denaturing for 15 sec at 95°C and annealing for 60 sec at 60°C. The primer sequences are listed in Table 1. The primers were designed using Primer Express 2.0 (Applied Biosystems, Foster City, CA, USA) to span intron-exon boundaries to avoid amplification of genomic DNA and selected to have compatible Tm values (59–61°C). A relative quantification method was used. Expression of mRNA of EP1, EP2, EP3, EP4, FP, PGT, total MRP4, and MRP4 variant 1 was normalized to that of the gene GAPDH. After each run, melting curve analysis (55–95°C) was performed to verify the specificity of the PCR reaction. All samples were tested in duplicate and each run included a template control. Baseline curves, melting curves, melting points, crossing points, slopes, and errors were monitored for each gene.

### 2.3. Immunohistochemical Probe

Endometrium was fixed in 10% formalin (Fisher Scientific, New Jersey, USA) and then embedded in paraffin. Serial tissue sections 4 *μ*m thick were rinsed in phosphate buffered saline (PBS) and treated with 3% hydrogen peroxide to block endogenous peroxidase activity. All antibodies were diluted in PBS containing 0.2% bovine serum albumin and 0.1% Tween 20. Sections were incubated for two hours at room temperature with the specific antibody (Cayman, Ann Arbor, USA). Rabbit polyclonal anti-human EP2 was diluted 1 : 800, and rabbit polyclonal antibodies directed against human EP1, EP3, EP4, FP, and PGT were all diluted 1 : 200. Rat monoclonal anti-human total MRP4 (Abcam, Cambridge, USA) was diluted 1 : 50. Sections were then held for 45 minutes at room temperature with peroxidase-conjugated goat anti-rabbit antibody (Jackson ImmunoResearch Laboratories, Inc., West Grove, USA) diluted 1 : 2000 or peroxidase-conjugated rabbit anti-rat antibody (Jackson ImmunoResearch Laboratories, Mississauga, Canada) diluted 1 : 500 and then for 40 minutes with the peroxidase substrate 3,3′-diaminobenzidine for 5 minutes at room temperature followed by rinsing in PBS, counterstaining with hematoxylin and mounting in Mowiol.

### 2.4. Statistical Analysis

Data that followed a normal (Gaussian) distribution were subjected to one-way analysis of variance (ANOVA) and Bonferroni's post hoc test for multiple comparisons, while data that were not normally distributed were analyzed using the Kruskal-Wallis test and Dunn's multiple comparison post hoctest for multiple comparisons. Comparison of the two groups was performed using the parametric unpaired *t*-test or the nonparametric Mann-Whitney test. All statistical analyses were performed using GraphPad Prism 5.0 Software (San Diego, CA, USA). Differences were considered to be statistically significant at *P* < 0.05.

## 3. Results

### 3.1. Expression of PGE2 and PGF2*α* Receptors and Transporters in Eutopic and Ectopic Endometria in the Diseased State

#### 3.1.1. Prostaglandins E2 and F2*α* Receptors

**Figure 1:**
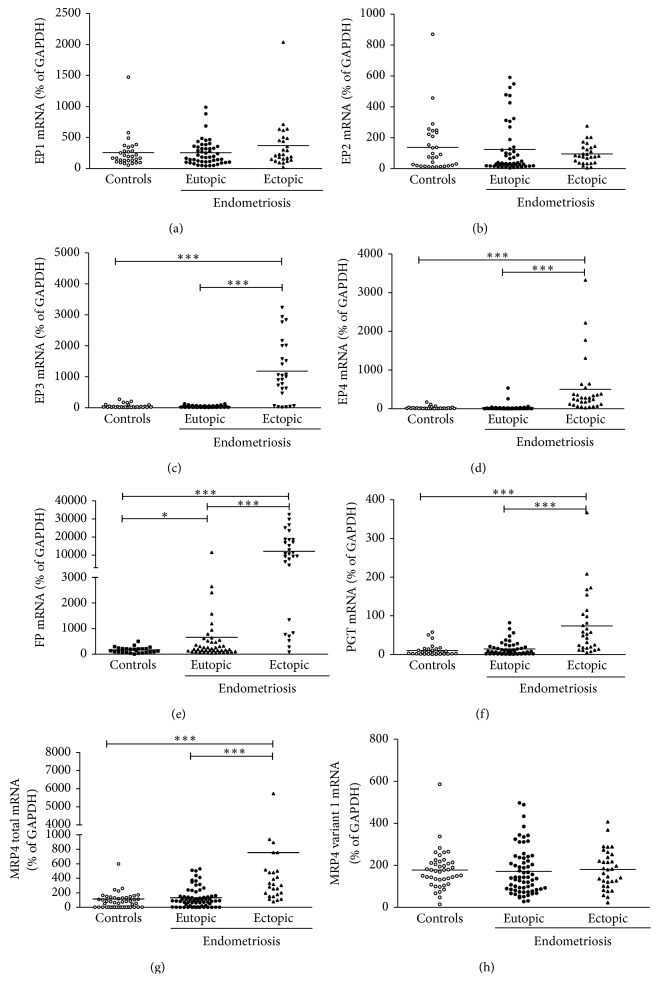
Expression of PGE2 and PGF2*α* receptors and prostaglandin transporters in endometrium, *n* = 30, 50, and 28, respectively, for healthy (control) subjects, eutopic tissue samples from endometriosis patients, and ectopic tissue samples. Total RNA was extracted and reverse-transcribed and mRNA was quantified by quantitative RT-PCR and normalized relative to GAPDH (internal control). (a) EP1, (b) EP2, (c) EP3, (d) EP4, (e) FP, (f) PGT, (g) total MRP4, and (h) MRP4 variant 1. The horizontal lines represent the mean for each set of data. ^*^ *P* < 0.05, ^**^ *P* < 0.01, and ^***^ *P* < 0.001.

**Figure 2:**
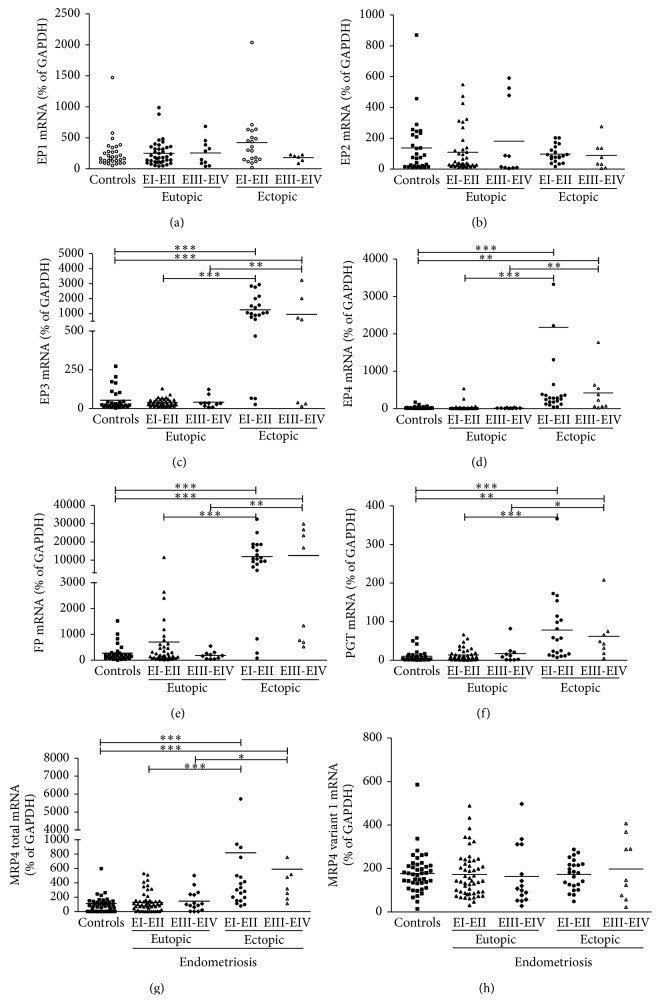
Expression of PGE2 and PGF2*α* receptors and prostaglandin transporters in endometrium at different stages of endometriosis, *n* = 30, 50, and 28, respectively, for healthy (control) subjects, eutopic tissue samples from patients with the disease, and ectopic tissue samples. Total RNA was extracted and reverse-transcribed and mRNA was quantified by quantitative RT-PCR and normalized relative to GAPDH (internal control). (a) EP1, (b) EP2, (c) EP3, (d) EP4, (e) FP, (f) PGT, (g) total MRP4, and (h) MRP4 variant 1. The horizontal lines represent the mean for each set of data. ^*^ *P* < 0.05, ^**^ *P* < 0.01, and ^***^ *P* < 0.001.

Neither EP1 nor EP2 was expressed differentially to any appreciable degree, whether the comparison was between endometriosis patients and healthy control patients, eutopic and ectopic endometrium in endometriosis patients (Figures 1(a) and 1(b)), endometriosis stages (Figures 2(a) and 2(b)), or the two phases of the menstrual cycle (Table 2).

Expression of EP3 was increased in ectopic endometrium of endometriosis patients (Figure 1(c)) and, as shown in Figure 2(c), in both stages I-II and stages III-IV. This effect was apparent in both secretory and proliferative phases of the menstrual cycle (Table 2). These effects were all significant at *P* < 0.001. It is worth mentioning that EP3 expression was increased significantly in eutopic tissue during the secretory phase even in healthy women (*P* < 0.05), but at an order of magnitude less than in ectopic tissue.

As was the case for EP3, EP4 expression was increased (*P* < 0.001) in ectopic endometrium (Figure 1(d)), although neither endometriosis stage (Figure 2(d)) nor phase of the menstrual cycle (Table 2) had any effect.

FP expression followed distinctive patterns in eutopic (*P* < 0.05) and ectopic (*P* < 0.001) endometria of endometriosis patients (Figure 1(e)). The increase was especially pronounced in ectopic endometrium (*P* < 0.001) at both disease stages (Figure 2(e)), during both phases of the menstrual cycle (Table 2). Expression in eutopic endometrium in endometriosis patients was significantly higher during the proliferative phase than during the secretory phase, while the difference between these patients and healthy subjects during the proliferative phase was only marginally significant (*P* = 0.18).

#### 3.1.2. Prostaglandins E2 and F2*α* Transporters

PGT expression was significantly increased (*P* < 0.001) in ectopic endometrium (Figure 1(f)) at stages I-II (*P* < 0.001) and III-IV (*P* < 0.01) of the disease (Figure 2(f)) and during both phases of the menstrual cycle (Table 2).

Expression of total MRP4 in ectopic endometrium was increased similar to PGT in endometriosis stages I-II and III-IV (Figures 1(g) and 2(g)) and during both phases of the menstrual cycle (Table 2). However, regulation of MRP4 is more complex because of its two transcriptional variants, namely, variant 1 (coding sequence: 120…4097, NM_005845.3) and a shorter variant 2 (coding sequence: 120…2699, NM_001105515), which encode distinctive functional proteins of different molecular mass. There was no noticeable effect of the disease on the expression of variant 1 (Figure 1(h)). The ratio of variant 1 to total MRP4 was significantly greater (*P* < 0.001) in eutopic endometrium in subjects with or without the disease (not shown). The meaning of this ratio is not yet known. In any case, it did not differ significantly.

### 3.2. Immunohistochemical Localization of PGE2 and PGF2*α* Receptors and Transporters in Eutopic and Ectopic Endometria in Endometriosis Patients and Healthy Subjects

**Figure 3:**
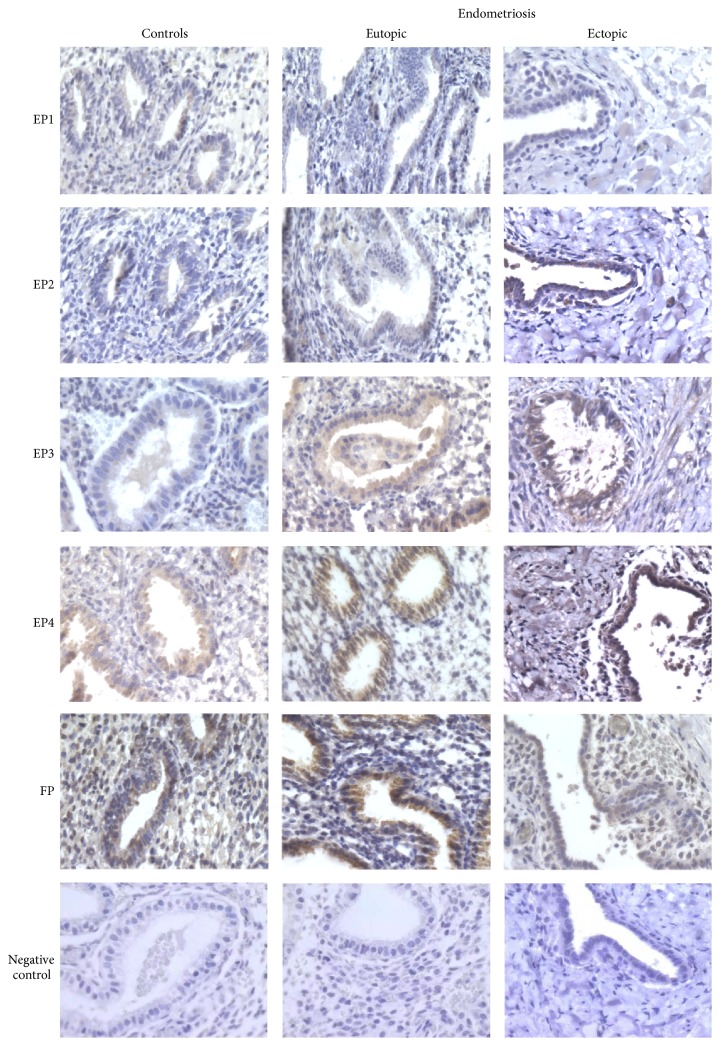
Representative immunohistochemical staining of EP1, EP2, EP3, EP4, and FP in endometrium of healthy women and in eutopic and ectopic endometrium of endometriosis patients. No staining was observed in control sections incubated without the primary antibody or with an equivalent concentration of goat IgG. The original magnification was 400x.

**Figure 4:**
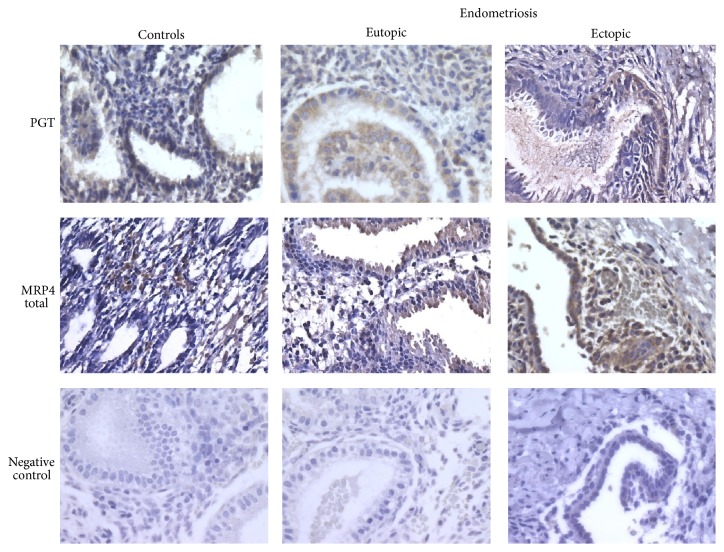
Representative immunohistochemical staining of PGT and total MRP4 in endometrium of healthy women and in eutopic and ectopic endometrium of endometriosis patients. No staining was observed in control sections incubated without the primary antibody or with an equivalent concentration of goat or rat IgG. The original magnification was 400x.

Cell membranes of ectopic endometriotic tissue and eutopic endometrium from endometriosis patients and from healthy subjects were examined using an immunohistochemical technique. Representative immunological staining of the receptors and transporters is shown in Figures 3 and 4. Staining of EP1 and EP2 in endometrial glands was weak. Clear staining of EP3, EP4, and FP was visible mainly in endometrial glands, but the adjacent stroma also appeared positive for these receptors. However, staining of FP was more intense in eutopic and ectopic endometrial tissues of endometriosis patients, whether in glandular cells or in surrounding stromal cells.

The pattern of PGT staining was similar in endometrial tissues from endometriosis patients and healthy subjects. Positive staining for MRP4 (total) was located mainly in stromal cells from control patients, but only in epithelial cells of eutopic endometrium from endometriosis patients. However, this staining was more intense in ectopic tissue, whether in glandular epithelial cells or in surrounding stromal cells.

## 4. Discussion

Prostaglandins E2 and F2*α* play major roles in the regulation of the cyclic changes of the endometrium and are also involved in diseases afflicting this tissue, in particular endometriosis. In this study, we showed that expression of mRNA encoding prostaglandin receptors EP3, EP4, and FP and of transporters PGT and MRP4 was increased in ectopic endometrium of women suffering from endometriosis. The expression of FP receptor was increased in both ectopic and eutopic endometrium of endometriosis patients, while expression of EP3 and EP4 was increased in ectopic endometrium only. No effect on expression of EP1 or EP2 receptors was observed. The menstrual cycle also had a significant effect, increasing EP3 receptor expression in the secretory phase both in the control group and in women with endometriosis. Although the cycle did not modulate overexpression of EP3, EP4, and FP in ectopic endometrium, it did appear to affect the eutopic endometrium of these patients, notably by causing decreased expression of FP in the secretory phase. This was not observed for any other receptor. While the increase in PGF2*α* during the secretory phase in healthy women is well documented, the regulation of PGE2 secretion during the menstrual cycle is less certain. Studies show that PGE2 level may increase during the secretory phase or the proliferative phase or may remain the same throughout the cycle [33–35]. Our data corroborate a previous observation of EP4 and FP expression increasing towards the end of the menstrual cycle and concomitant with the withdrawal of progesterone and sloughing of the functional layer of the endometrium, peaking during the mid-late proliferative phase and not in the secretory phase, coincident with an elevation in the expression of PGT [36]. In contrast, changes in EP3 expression across the menstrual cycle have not been noted previously [37–39].

The specific roles played by PGE2 and PGF2*α* in modulating reproductive physiology have been demonstrated using mice deficient in the corresponding receptors [40]. The most striking observations have been made using FP receptor and EP3 receptor knockout mice. It has thus been shown that the FP receptor is indispensable in female reproduction and that its ablation results in loss of parturition [41]. Studies of mice lacking individual prostaglandin receptors EP1–4 suggested strongly that EP3 was the principle receptor mediating pain [42, 43]. In addition, several* in vitro* studies demonstrated that expression of aromatase (an enzyme involved in the synthesis of estrogens) may be regulated through EP3 [44]. On the other hand, binding of PGE2 to the EP3 receptor regulates vascular function-dysfunction in ocular tissues and promotes vitreal neovascular diseases such as ischemic retinopathy [45] and also transcriptional upregulation of fibroblast growth factor 9 [46]. Although little is known about the angiogenic potential of other prostaglandin receptors, increased levels of EP4 and FP have been reported in perivascular cells in endometrial adenocarcinomas [27, 47]. More recent studies have demonstrated that selective blockade of EP4 signaling inhibits proliferation and adhesion of human endometriotic epithelial and stromal cells through suppression of integrin-mediated mechanisms [48–50]. It has also been shown that anomalies in cell adhesion, morphology, and proliferation can occur after binding of ligands to EP4 and FP or activation of downstream signaling pathways such as MAPK and PI3K [47, 51–53]. Our results suggest that the specific increase in expression of these receptors is not insignificant and that it may contribute to the principal symptoms associated with endometriosis. Very few authors have studied the role of MRP4 in endometriosis [54]. Increased MRP4 expression has been shown in malignant prostate tissue [55], in acute myeloid leukemia [56], and in colorectal neoplasia [57], while increased PGT expression has been associated with epithelial malignancy [58]. Our results show, for the first time, increased expression of both prostaglandin transporters in endometriotic tissues. We observed significant increases of PGT expression in eutopic endometrium during the proliferative phase of the menstrual cycle both in healthy women and in patients with endometriosis as well as in ectopic endometrium. As reported previously [54, 59], we also observed significantly increased expression of total MRP4 in ectopic tissue while MRP4 variant 1 showed no noticeable change. Recently described as a prostaglandin efflux transporter, MRP4 was expressed at much the same level throughout the menstrual cycle. Based on these results, we suggest that the observed overexpression of MRP4 is most likely due to variant 2 and not to the combination of the two variants, as is often reported. The increases in both transporter molecules appear concomitant, specifically in ectopic lesions. Although their actions seem to be different, these transporters might work in concert to improve prostaglandin dispatching.

Immunohistochemistry experiments largely support the mRNA expression results. In eutopic endometrium, PGE2 and PGF2*α* receptors and transporters were found at greater abundance in glandular epithelial cells than in stromal cells, consistent with increased epithelial MRP4 expression demonstrated in endometriosis [54] and cancer [55]. Furthermore, staining of PGT, FP, EP3, and EP4 in ectopic endometrial tissue was intense, as reported previously [60, 61]. Low levels of EP1 and EP2 were also found in the glandular epithelium and in ectopic tissue. Our results corroborate those of Arosh et al. [62], who reported that EP2 staining in the endometrium of cattle was expressed mainly in glandular epithelial cells.

Elevated MRP4 and PGT expression, particularly in epithelial glandular cells, may result in increased availability of PGE2 and PGF2*α*, which through engagement of their EP3, EP4, or FP receptors (also elevated in endometriosis) may activate intracellular signals such as the diacylglycerol or cyclic AMP pathways. Aberrant transport and signaling by prostaglandin receptors in the endometrium [47, 63] as observed in the present study therefore might promote uterine pathologies such as endometriosis. Although the modifications observed in the eutopic endometrium of endometriosis patients appear slight, they nevertheless affect sensitivity to PGE2 and PGF2*α* and thus disrupt normal function. The proinflammatory environment of the peritoneal cavity of women with endometriosis induces significant overexpression of the majority transporters and receptors required to regulate PGE2 and PGF2*α*. In addition to having a role in the pathogenesis of the disease, this could also disrupt the entire female reproductive tract. Since the female genitalia bathe in peritoneal liquid [64], an increased level of PGE2 and PGF2*α* could act on the whole system and thereby influence the reproductive process.

Although qualitative, immunohistochemistry has limitations that should not be overlooked. Immunohistochemical confirmation of mRNA expression reinforces the importance of our observations, especially in view of the inevitable variability associated with categorizing of clinical symptoms and/or staging of patients. In conclusion, this study reveals for the first time that endometriosis can affect the regulation of PGE2 and PGF2*α* activity at the points of reception on the cell surface and transport into the target cells.

*Capsule.* Eutopic and ectopic endometria of endometriosis patients display distinct anomalies in levels of expression of prostaglandins receptivity and uptake factors.

## Figures and Tables

**Table 1:** List of primers used for qRT-PCR.

Gene	Forward	Reverse
EP1	5′-ATGGTGGGCCAGCTTGTC-3′	5′-GCCACCAACACCAGCATTG-3′
EP2	5′-TCCTTGCCTTTCACGATTT-3′	5′-AGAGCTTGGAGGTCCCATT-3′
EP3	5′-TGGTCTCCGCTCCTGATAA-3′	5′-TGCATTCTTTCTGCTTCTCC-3′
EP4	5′-TGCTCTTCTTCAGCCTGTCC-3′	5′-GAGCTACCGAGACCCATGTT-3′
FP	5′-TCTGGTCTGTGCCCACTTC-3′	5′-GACTCCAATACACCGCTCAAT-3′
PGT	5′-CTGGTGGATTTCATTAAACGG-3′	5′-GGCTGCTGAGGTGCCATAC-3′
MRP4 total	5′-AAAGTGCCAAAGTAATCCAGC-3′	5′-GTTCAAAGCCACAGAATCCA-3′
MRP4 variant 1	5′-CGGGCATACAAAGCAGAA-3′	5′-GGACCCAAAGGCAACG-3′

**Table 2:** Effect of endometriosis and menstrual cycle phase on expression of prostaglandin receptors and transporters in endometrium, based on real-time PCR and normalized relative to GAPDH mRNA as mean % ± SD. Also, *n* = 30, 50, and 28, respectively, for healthy (control) subjects, eutopic tissue samples from endometriosis patients, and ectopic tissue samples.

Gene	Controls (mean ± SEM)	Endometriosis	
		Eutopic (mean ± SEM)	Ectopic (mean ± SEM)
*EP1 *			
Proliferative phase	317,2 ± 100,5	307,0 ± 42,13	307,3 ± 54,11
Secretory phase	209,5 ± 35,33	200,2 ± 37,57	411,3 ± 128,9

*EP2 *			
Proliferative phase	57,90 ± 17,46	66,60 ± 22,24	71,74 ± 14,89
Secretory phase	198,2 ± 51,72^†^	179,5 ± 38,82^†^	112,6 ± 17,9

*EP3 *			
Proliferative phase	28,55 ± 6,652	29,76 ± 3,444	1021 ± 249,3^∗∗∗+++^
Secretory phase	73,94 ± 20,06^†^	48,35 ± 6,463^††^	1310 ± 276,1^∗∗∗+++^

*EP4 *			
Proliferative phase	34,38 ± 12,67	46,82 ± 22,68	380,2 ± 136,5^∗∗∗+^
Secretory phase	21,94 ± 6,654	10,47 ± 1,546	583,4 ± 205,2^∗∗∗+++^

*FP *			
Proliferative phase	237,5 ± 31,18	1059 ± 497,2	9213 ± 1947^∗∗∗+++^
Secretory phase	97,27 ± 18,26^††^	229,6 ± 61,22^††^	14268 ± 2742^∗∗∗+++^

*PGT *			
Proliferative phase	16,04 ± 4,058	21,74 ± 3,682	56,42 ± 14,73^∗∗+^
Secretory phase	5,964 ± 3,326^††^	6,760 ± 2,736^†††^	88,69 ± 25,28^∗∗∗+++^

*MRP4 total *			
Proliferative phase	142,9 ± 63,47	97,05 ± 15,51	591,5 ± 227^∗∗+^
Secretory phase	80,93 ± 16,17	154,7 ± 25,77	931,2 ± 386,5^∗∗∗+++^

*MRP4 variant 1 *			
Proliferative phase	160,9 ± 15,28	157,8 ± 18,82	194,2 ± 24,08
Secretory phase	196,2 ± 24,35	178,8 ± 18,99	164,7 ± 20,93

Note: ^**^ *P* < 0.01 and ^***^ *P* < 0.001 versus the control group; ^+^ *P* < 0.05 and ^+++^ *P* < 0.001 versus the eutopic group; ^†^ *P* < 0.05 and ^††^ *P* < 0.01 versus expression in the corresponding proliferative phase; ^†††^ *P* < 0.001.
